# Ecological Momentary Assessment of Cognition in Clinical and Community Samples: Reliability and Validity Study

**DOI:** 10.2196/45028

**Published:** 2023-06-02

**Authors:** Shifali Singh, Roger Strong, Irene Xu, Luciana M Fonseca, Zoe Hawks, Elizabeth Grinspoon, Lanee Jung, Frances Li, Ruth S Weinstock, Martin J Sliwinski, Naomi S Chaytor, Laura T Germine

**Affiliations:** 1 McLean Hospital Belmont, MA United States; 2 Harvard Medical School Boston, MA United States; 3 Elson S Floyd College of Medicine, Washington State University Pullman, WA United States; 4 Programa Terceira Idade (PROTER, Old Age Research Group), Department and Institute of Psychiatry, University of São Paulo School of Medicine Sao Paolo Brazil; 5 Department of Medicine State University of New York (SUNY) Upstate Medical University Syracuse, NY United States; 6 Department of Human Development and Family Studies The Pennsylvania State University State College, PA United States; 7 Center for Healthy Aging Pennsylvania State University State College, PA United States

**Keywords:** ecological momentary assessment, cognition, digital neuropsychology, remote assessment, digital technology, type 1 diabetes, teleneuropsychology, reliability, validity, cognitive functioning, psychological, physiological, glucose, community

## Abstract

**Background:**

The current methods of evaluating cognitive functioning typically rely on a single time point to assess and characterize an individual’s performance. However, cognitive functioning fluctuates within individuals over time in relation to environmental, psychological, and physiological contexts. This limits the generalizability and diagnostic utility of single time point assessments, particularly among individuals who may exhibit large variations in cognition depending on physiological or psychological context (eg, those with type 1 diabetes [T1D], who may have fluctuating glucose concentrations throughout the day).

**Objective:**

We aimed to report the reliability and validity of cognitive ecological momentary assessment (EMA) as a method for understanding between-person differences and capturing within-person variation in cognition over time in a community sample and sample of adults with T1D.

**Methods:**

Cognitive performance was measured 3 times a day for 15 days in the sample of adults with T1D (n=198, recruited through endocrinology clinics) and for 10 days in the community sample (n=128, recruited from TestMyBrain, a web-based citizen science platform) using ultrabrief cognitive tests developed for cognitive EMA. Our cognitive EMA platform allowed for remote, automated assessment in participants’ natural environments, enabling the measurement of within-person cognitive variation without the burden of repeated laboratory or clinic visits. This allowed us to evaluate reliability and validity in samples that differed in their expected degree of cognitive variability as well as the method of recruitment.

**Results:**

The results demonstrate excellent between-person reliability (ranging from 0.95 to 0.99) and construct validity of cognitive EMA in both the sample of adults with T1D and community sample. Within-person reliability in both samples (ranging from 0.20 to 0.80) was comparable with that observed in previous studies in healthy older adults. As expected, the full-length baseline and EMA versions of TestMyBrain tests correlated highly with one another and loaded together on the expected cognitive domains when using exploratory factor analysis. Interruptions had higher negative impacts on accuracy-based outcomes (β=−.34 to −.26; all *P* values <.001) than on reaction time–based outcomes (β=−.07 to −.02; *P*<.001 to *P*=.40).

**Conclusions:**

We demonstrated that ultrabrief mobile assessments are both reliable and valid across 2 very different clinic versus community samples, despite the conditions in which cognitive EMAs are administered, which are often associated with more noise and variability. The psychometric characteristics described here should be leveraged appropriately depending on the goals of the cognitive assessment (eg, diagnostic vs everyday functioning) and the population being studied.

## Introduction

### Background

Traditional neuropsychological assessment methods typically rely on single time point assessments that capture only a snapshot of an individual’s cognitive functioning. The advent of novel technologies can facilitate more comprehensive data capture for understanding cognitive functioning, including the real-time, high-frequency, and context-informed capture of cognitive data [1]. High-frequency data captured over time can provide rich data sets for understanding within-person cognitive variability while closing many key gaps in neuropsychological research and in the practice of neuropsychology. For example, evaluating cognition in the context of environmental, psychological, and physiological factors can aid in better ascertaining the degree of variability in day-to-day performance, which may have considerable functional impact, whereas a single, in-person assessment may poorly reflect everyday cognitive functioning if a patient is not experiencing their usual degree of cognitive symptoms when being tested (whether owing to chance or *rising to the occasion* for the test) or is being tested at a nonoptimal time of day [2]. Thus, traditional evaluations completed in the laboratory or clinic, which often cannot account for these factors, might misrepresent cognitive and functional capabilities [3,4]. This limitation contributes to poorer ecological validity and potentially limits the between- and within-person reliabilities of the results gleaned from neuropsychological testing [5,6]. It can also be invalidating to individuals who appear to perform well under optimal conditions yet experience cognitive difficulty in everyday life. These patients may be told that they are performing within normal limits yet continue to experience cognitive difficulty in their daily lives. Furthermore, in limiting our assessments to a single time point, we may not be able to adequately capture the associations between poorer cognition and modifiable factors, such as sleep or time of day, which may differentially impact cognition. By capturing multiple time points of assessment, we gain access to richer information about an individual’s cognition and ways specific contextual factors might be impacting their functioning in everyday life outside the clinic.

### Cognitive Ecological Momentary Assessment

Cognitive ecological momentary assessment (EMA) has evolved to address the limitations of single time point assessment through high-frequency assessment using brief cognitive measures delivered within an individual’s naturalistic environment. This approach permits a better estimation of mean performance [6] and performance variability [2], as well as the potential for greater ecological validity and generalizability because performance is assessed within an individual’s unique context instead of an artificially controlled laboratory setting [7].

In clinical samples, the application of cognitive EMA has further advantages beyond enabling denser data collection. Because of the COVID-19 pandemic, individuals have become more reluctant to attend in-person clinic visits; this is especially true for patients with chronic or comorbid medical and psychiatric conditions, who are more likely to experience chronic or severe disease outcomes [8]. Since March 2020, neuropsychological evaluations have often been conducted remotely, in-person, or using a hybrid in-person or remote model [9]. Cognitive EMA may be preferable or complementary to the current method of neuropsychological test administration in that it enables valid and reliable remote test administration without supervision; this remains true even in older adults, who demonstrate strong adherence to cognitive EMA when provided with support [10]. Cognitive EMA also allows for more measurement distribution, which allows for “averaging out” a discrepant or outlying performance while also quantifying performance instability as a potentially clinically relevant signature [11].

Although EMA as a scientific methodology is not new (eg, Smyth and Stone [12] and Shiffman et al [13]), it has only recently been used in neuropsychology to assess fluctuations in cognitive status. Basic validation research in this area has primarily focused on measurement burst designs for evaluating longitudinal cognitive change associated with aging [6] or testing specific hypotheses (eg, Hyun et al [14]). This study aimed to extend current research by characterizing the reliability and validity of ultrabrief cognitive EMA in both a clinical sample with high expected cognitive variability (type 1 diabetes [T1D]) and a community sample with lower, normative levels of cognitive variability.

### People With T1D

T1D is a chronic autoimmune disease characterized by elevated blood glucose levels resulting from the loss of insulin-producing beta cells in the pancreas. The management of the disease requires intensive insulin therapy using multiple daily insulin injections or an insulin pump. Fluctuations in glucose concentrations, including frequent episodes of hyperglycemia (high blood glucose) and hypoglycemia (low blood glucose), are common, and studies have linked them to short-term fluctuations in cognitive and psychological status (eg, Brands et al [15] and Mõttus et al [16]). Although capturing ecologically valid cognitive variation can be advantageous in any context, it is particularly useful for characterizing cognition in this population, in which known physiological factors can impact cognitive performance in the timescale of minutes, hours, and days. Therefore, using cognitive EMA in a sample with T1D permits the evaluation of reliability and validity in a sample with higher cognitive variability due to the impact of regular physiological changes.

### This Study

This study reports on the psychometric properties, including reliability and validity, of a set of ultrabrief cognitive EMA measures in a sample of adults with T1D as well as in a community sample. The primary goal of this study was to determine whether the cognitive test scores measured using cognitive EMA show good evidence for reliability (between-person and within-person, explained in detail in the subsequent section) and construct validity in both of these samples.

## Methods

### Participants

Participants were recruited from 2 sources for this study. First, adults with T1D were recruited from 4 endocrinology clinics across the country as part of an observational study (the GluCog study), which aimed to characterize the relationship between glycemic excursions and cognitive variability in adults diagnosed with T1D. Second, participants from the community were recruited through links on *TestMyBrain* (TMB [17]; the authors’ digital research platform) as part of a separate observational study (the MoodCog study) oriented around understanding the associations between fluctuations in mood and cognition in healthy, nonpsychiatric participants.

In the GluCog study, 203 participants were enrolled, and 198 (97.5%) met the criteria for inclusion in the final analytic data set based on the completion of at least 50% of the EMAs. All participants had a diagnosis of T1D. In this sample, we did not exclude individuals or time points based on glycemic excursions (episodes of low or high blood glucose, data on which were collected passively using continuous glucose monitoring), as these are the primary expected sources of increased cognitive variability. In the MoodCog study, 156 participants were enrolled, and 128 (82.1%) completed at least 50% of the EMAs. Table 1 lists further inclusion and exclusion criteria for each study. Table S1 in Multimedia Appendix 1 provides further details on participant compliance. The exclusion criteria were decided as part of the study protocol before this analysis and have been described in our published protocol for the T1D study [18] as well as in a paper now published using data from the community sample [19].

**Table 1:** Inclusion and exclusion criteria for the GluCog and MoodCog groups.

	GluCog	MoodCog
Inclusion criteria	Age of ≥18 at the time of initial assessment Fluency in English 24-hour access to a smart phone with reliable internet access Diagnosis of type 1 diabetes with >1 year duration	Age of ≥18 at the time of initial assessment Fluency in English 24-hour access to a smart phone with reliable internet access
Exclusion criteria	Inability to complete cognitive EMA^a^ during the study period (eg, because of working night shifts, planned travel across time zones, or an occupation that does not reliably allow breaks) Disabilities that would substantially interfere with the study protocol (eg, motor or visual impairment and a history of head trauma) Current psychiatric condition or medical condition or treatment that may interfere with the study protocol (eg, substance use disorder, chemotherapy, inpatient psychiatric admission, and a diagnosis of dementia), obtained via medical records	Inability to complete cognitive EMA during the study period (eg, because of working night shifts, planned travel across time zones, or an occupation that does not reliably allow breaks) Disabilities that would substantially interfere with the study protocol (eg, motor or visual impairment and a history of head trauma) Current psychiatric condition or medical condition or treatment that may interfere with the study protocol (eg, substance use disorder, chemotherapy, inpatient psychiatric admission, and a diagnosis of dementia), obtained via self-report

^a^EMA: ecological momentary assessment.

### Procedure

**Figure 1:**
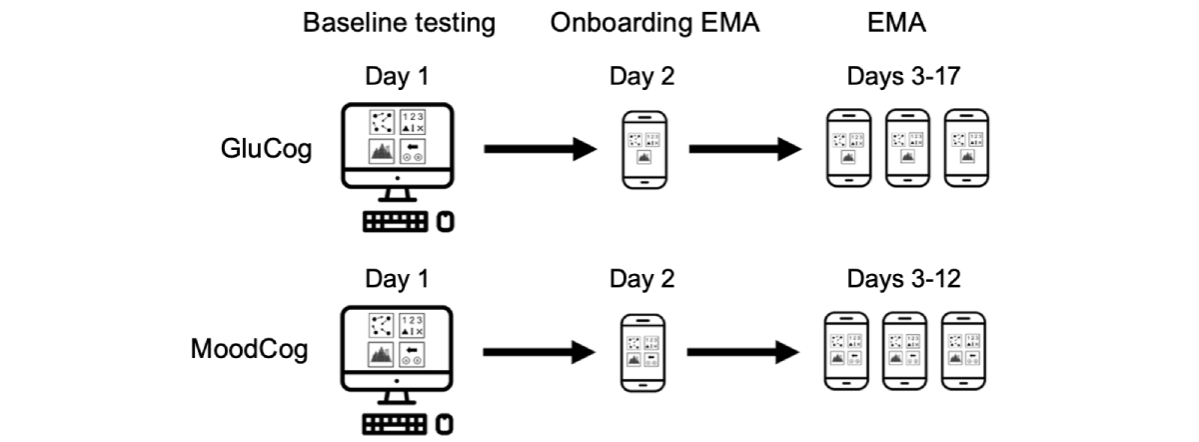
Testing schedules for the GluCog and the MoodCog groups. EMA: ecological momentary assessment.

**Figure 2:**
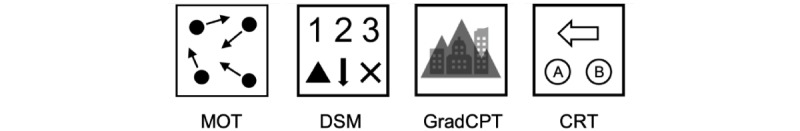
Illustrations of TestMyBrain cognitive tests administered in the GluCog and MoodCog groups. The participants in the GluCog sample did not complete Choice Reaction Time (CRT). DSM: Digit Symbol Matching; GradCPT: Gradual Onset Continuous Performance Test; MOT: Multiple Object Tracking.

Figure 1 illustrates the assessment schedules for the GluCog and MoodCog studies. Within the 2 groups, all cognitive tests were performed at each measurement occasion. On the first day of the study, the participants completed a baseline battery of cognitive tests, including standard, full-length versions of the tests that were completed as part of cognitive EMA (Figure 2). On the second day, the participants completed a single onboarding cognitive EMA on their smartphone to familiarize themselves with the shorter EMA versions of the tests and confirm their understanding of the study procedures. Data from the onboarding EMA were excluded from our analytic data set. On the third day and for the next 10 days (MoodCog) or 15 days (GluCog), the participants received texts on their phone containing links to a short battery of cognitive EMA measures 3 times a day: once in the morning, once in the afternoon, and once in the evening. The texts were sent on a pseudorandom schedule to capture a range of times across the day. Upon receiving the text, the participants had up to 30 minutes to complete the cognitive EMA. The participants received a reminder text 20 minutes after the first text to encourage completion. The GluCog sample completed 15 days of cognitive EMAs (a total of 45 cognitive EMAs), whereas the MoodCog sample completed 10 days of cognitive EMAs (a total of 30 cognitive EMAs). The test trials and test orders were counterbalanced across cognitive EMA time points, although each participant received the same test trials and test orders at any given time point. All ultrabrief tests were developed for brief administration using an iterative test development procedure to ensure that the test formats, trials, and lengths were adequate for capturing cognitive functioning at individual time points [1]. Psychometric characteristics were evaluated for each alternate form to ensure that they had similar reliability and distributional properties. The psychometric characteristics of the individual, ultrabrief TMB tests used in this study have also been previously described, although within the context of glycemic variability in a sample of individuals with T1D [18].

### Measures

#### TMB Multiple Object Tracking

The TMB Multiple Object Tracking (MOT) task assesses visuospatial attention and visual working memory [20,21]. On each trial of the MOT task, a subset of black dots is designated as targets by turning green before turning back to black and moving around the screen among nontarget dots. The participants are asked to keep track of those target dots during 5 seconds of movement; once the dots stop moving, the participants are asked to select the dots originally designated as targets.

The full-length version has 18 test trials, 6 trials each at the target set sizes of 3, 4, and 5 dots, and takes 6 minutes to complete. Within each set size, the dots move relatively slowly on the first trial and increase in speed in each additional trial. Before beginning the test trials in the full-length assessment, the participants complete 2 practice trials, one at set size 2 and another at set size 3. In the ultrabrief EMA version, there are 6 test trials, all with a target set size of 5 dots and no practice trials, and it takes 2 minutes to complete. The speed of the dots increases in each successive trial. The score represents the percentage of target dots correctly identified during the test trials.

#### TMB Digit Symbol Matching

The TMB Digit Symbol Matching (DSM) test, adapted from the Wechsler Adult Intelligence Scale-Third Edition (WAIS-III) [22], assesses processing speed [23,24]. The participants are presented with 6 symbols, each of which is paired with a single digit between 1 and 3 (ie, 2 symbols lined up vertically under each digit); these digit-symbol pairings remain visible throughout the duration of the test. Individual probe symbols are sequentially presented above these pairings and remain visible until the participants make a response. The participants respond by selecting the corresponding digit (using the keyboard or touchscreen) as quickly as possible for either 90 seconds in the full-length version or 30 seconds in the ultrabrief EMA version. The full-length version starts with 3 practice trials. The score for the DSM test is the median reaction time of correct responses to test probes (the number of items presented varies across test administrations).

#### TMB Gradual Onset Continuous Performance Test

The TMB Gradual Onset Continuous Performance Test (GradCPT) assesses sustained attention, cognitive control, and response inhibition [25,26]. The participants view a sequence of grayscale images of cities and mountains, with the images gradually transitioning from one to the next every 800 milliseconds. The participants are instructed to tap the screen when images of cities are presented (80% of the trials) and withhold from tapping the screen when images of mountains are presented (20% of the trials). The total duration of the image sequence is 240 seconds for the full-length test and 60 seconds for the ultrabrief EMA. The score represents the participants’ discrimination ability (d-prime), a signal detection measure based on response accuracy corrected for response bias.

#### TMB Choice Reaction Time

The TMB Choice Reaction Time (CRT) assesses processing speed, cognitive inhibition, and cognitive control [27,28]. On each trial of the CRT, the participants view 3 vertically aligned squares, each of which contains a color (blue or yellow) and an arrow (pointing either left or right). Of the 3 squares, 1 is always of a different color from the other 2; the participants are instructed to indicate the direction of the arrow in the differently colored square as quickly as possible by clicking or tapping. The participants complete 4 practice trials and 30 test trials in the full-length test, for a duration of 3 minutes, and 24 test trials in the ultrabrief EMA test, for a duration of 1 to 2 minutes. The CRT was administered as an EMA measure only for the MoodCog group. The score represents the median reaction time in correctly answered test trials.

#### TMB Vocabulary Test (Baseline Only)

The TMB Vocabulary Test assesses crystalized cognitive ability and word knowledge, which have been shown to positively correlate with age [24], educational attainment, and Wechsler Adult Intelligence Scale-Fourth Edition (WAIS-IV) vocabulary performance [23]. On each trial of the test, the participants view a target word and are asked to select which of 5 response options is closest in meaning to that target word. The Vocabulary Test is only administered at the baseline and includes 1 practice trial and 30 test trials. The score represents the percentage of correctly answered test trials. It takes approximately 4 minutes to complete.

#### Interruptions Survey (EMA Only)

At the end of each cognitive EMA, the participants were asked, “Did anything interrupt you during this assessment?” To this, the participants responded with “yes” or “no,” and then they were asked to provide a brief explanation for their response.

### Statistical Analyses

#### Cognitive EMA Reliability

For each outcome measured using cognitive EMA, we calculated two different metrics of reliability [6]: (1) between-person reliability (the ratio of variance in scores attributable to differences in individuals, relative to within individuals) and (2) within-person reliability (the proportion of total variance in scores attributable to differences across measurement occasions, relative to within measurement occasions). To allow the calculation of these 2 measures of reliability, each cognitive test outcome was separately computed for the even and odd trials of each cognitive EMA, with some slight variance in this procedure depending on the test (ie, for TMB GradCPT, we looked at even or odd separately for cities and mountains, and for TMB MOT, we split trials 1, 4, and 6 and 2, 3, and 5 to equate for difficulty). The *mlr* function (*mlr* stands for multilevel reliability, which finds and plots reliability and generalizability coefficients for multilevel data) of the *psych* package [29,30] in R (R Foundation for Statistical Computing) was then used to compute between-person reliability (labeled *RkRn* in the *mlr* output) and within-person reliability (labeled *Rcn* in the *mlr* output), using unconditional multilevel mixed models to predict performance on each half of each EMA, with the random effects of EMA number nested within participants. Of note, we believe that this was the most consistent approach to calculating reliability, given that in MOT, trials are not at the same level of difficulty; in GCPT, we cannot compute a score for an individual’s trials; and in DSM, there are not an equal number of trials on each EMA version. Thus, we could not compute within-trial variance from the scores from each trial (calculating within-person reliability as a function of the number of trials administered) in a way that would be consistent across tests.

Previous work has indicated that reliability estimates will often reach an asymptote after a certain number of EMAs. Therefore, we also estimated the number of EMAs needed to achieve maximum (or near maximum) reliability, between person and within person, for each cognitive test.

#### Cognitive EMA Validity

We evaluated construct validity using factor analysis within both samples. For both samples, we conducted an exploratory factor analysis with direct oblimin rotation (to allow correlations between factors) using the *psych* package’s *fa*() function [29] and used a manifest average.

We also evaluated the criterion validity by examining correlations with age for all cognitive tests. On the basis of previous work, it was expected that scores on speed-based measures (CRT and DSM) and visuospatial working memory (MOT) would exhibit sharp declines with age [24,27] and that sustained attention would show minimal declines with age [25]. Although vocabulary was not included in cognitive EMA, we included scores on the TMB Vocabulary Test as a comparison condition, as vocabulary scores typically improve with age, even as other cognitive skills decline [24].

#### Other Analyses: Self-reported Interruptions

We aimed to better understand how interruptions (the participants indicated “yes” or “no” to the question about whether there were any interruptions) affected performance; as a validity indicator, we expected that greater interruptions would be associated with poorer performance on cognitive tests. To assess the impact of interruptions on EMA task performance, each cognitive performance outcome was first standardized (using *z* scores) across all EMAs, separately for each sample. These standardized cognitive EMA outcomes were then predicted using multilevel mixed models, with fixed effects of interruptions (yes or no), gender, and age, and a random intercept of participants; this approach allowed us to determine the unique contribution of interruptions to performance on each outcome measured using cognitive EMA.

### Ethics Approval

The study protocol was approved by the institutional review board (IRB) associated with each study (GluCog: Jaeb Center for Health Research IRB Protocol 2023P000083; MoodCog: Mass General Brigham IRB Protocol 2019P000538). All participants provided informed consent to participate in this study.

## Results

### Demographic Characteristics

The demographic characteristics for both groups are presented in Table 2. Compared with the MoodCog participants, the GluCog participants were significantly older (*t*_328_=5.21, *P*<.001) and more likely to be male (GluCog + MoodCog, N=326; χ*^2^*_2_=32.6, *P*<.001). The GluCog participants completed an average of 86.1% (SD 10%) of the 45 cognitive EMAs assigned to them, and the MoodCog participants completed an average of 84.5% (SD 10.2%) of the 30 cognitive EMAs assigned to them. We excluded EMAs that did not meet the quality control criteria (refer to Table S2 in Multimedia Appendix 1 for the quality control criteria and number of EMAs excluded).

**Table 2:** GluCog and MoodCog participant demographic characteristics.

Characteristic			GluCog (n=198)		MoodCog (n=128)		*P* value	
EMA^a^ completion (%), mean (SD)			86.09 (9.97)		84.47 (10.15)		.15^b^	
Age (years), mean (SD)			45.69 (15.58)		36.77 (14.66)		<.001^b^	
**Gender, n (%)**								<.001^b^
	Woman	54.04 (107)		77.27 (102)				
	Man	44.95 (89)		17.42 (23)				
	Nonbinary	0.51 (1)		5.30 (7)				
**Race, n (%)**								.07^c^
	Asian	1.01 (2)		9.85 (13)				
	Black	7.07 (14)		4.55 (6)				
	Native American	1.01 (2)		0.76 (1)				
	Pacific Islander	0.51 (1)		0 (0)				
	White	88.89 (176)		81.06 (107)				
	Missing or other	4.04 (8)		8.33 (11)				
**Hispanic or Latino, n (%)**								.70^c^
	Yes	6.57 (13)		8.33 (11)				
	No	92.93 (184)		89.39 (118)				
	Missing	0.51 (1)		2.27 (3)				
Education (years), mean (SD)			15.62 (1.9)		15.95 (2.41)		.17^b^	

^a^EMA: ecological momentary assessment.

^b^Welsh independent samples 2-tailed *t* test.

^c^Pearson chi-square test. As some racial categories had too few participants to ensure accuracy when estimating group differences, the race categories were collapsed into a “White” group and a “non-White” group when conducting the chi-square test.

### Descriptive Characteristics and Reliability of Cognitive EMA

Table 3 shows the means and SDs of the performance on the full-length baseline and ultrabrief EMA versions of each cognitive test, separately for the GluCog and MoodCog groups. As expected, performance variability was significantly higher among the GluCog participants than among the MoodCog participants on the TMB DSM task (*t*_321_=5.77, *P*<.001). Variability on the TMB MOT and TMB GradCPT tasks did not significantly differ between the 2 groups (TMB MOT: *t*_256.7_=−1.17, *P*=.25; TMB GradCPT: *t*_272.5_=1.6, *P*=.11).

Table 3 also shows the between- and within-person reliabilities for each outcome measured using cognitive EMA. The traditional form of reliability reported for neuropsychological tests is a kind of between-person reliability, which evaluates the consistency of the differences in scores between individuals. Both groups demonstrated very high between-person reliability for both the full-length baseline and ultrabrief EMA versions of cognitive tests. Given the large number of test time points and known between-person reliability of the original tests, these very high levels of between-person reliability were in line with expectations. Within-person reliability is a separate form of reliability that assesses the degree to which the differences between assessments are large or small relative to the differences among items and trials within a single assessment. Thus, within-person reliability depends on variability across assessments and is minimized when there is little to no variability in test scores within individuals. Within-person reliability varied substantially across tests in both groups. Within-person reliability was lower for accuracy-based scores (MOT and GradCPT) than for reaction time–based scores (DSM and CRT) in both groups. Of the tests that were included in both studies, MOT had the lowest within-person reliability in both groups, and DSM had the highest within-person reliability in both groups. This suggests that reaction time–based measures, such as DSM, may be most useful for examining variability over time within participants. The magnitude of within-person reliability for these tests was comparable with that observed in other studies that used cognitive EMA (eg, Sliwinski et al [6]).

**Table 3:** Descriptive statistics and reliabilities of the performance on the full-length baseline and ultrabrief ecological momentary assessment (EMA) versions of the cognitive tests, separately for the GluCog and MoodCog groups.

Outcome			GluCog							MoodCog				
			Value, mean (SD)		BPR^a^		WPR^b^		Value, mean (SD)			BPR		WPR
**Baseline versions**														
	MOT^c^ accuracy (%)	73.8 (10.3)		0.89		—^d^		80.2 (8.8)			0.84		—	
	DSM^e^ medianRTc^f^ (ms)	1070 (344)		0.98		—		843 (203)			0.99		—	
	GradCPT^g^ d-prime^h^	2.45 (0.84)		0.86		—		2.81 (0.74)			0.82		—	
	CRT^i^ medianRTc (ms)	1010 (363)		0.98		—		876 (294)			0.98		—	
**EMA versions**														
	MOT accuracy (%)	68.5 (8.8)		0.98		0.20		73.9 (8.7)			0.97		0.28	
	DSM medianRTc (ms)	968 (235)		0.99		0.65		817 (140)			0.98		0.72	
	GradCPT d-prime	2.45 (0.67)		0.98		0.54		2.78 (0.49)			0.95		0.55	
	CRT medianRTc (ms)	—		—		—		720 (109)			0.98		0.80	

^a^BPR: between-person reliability.

^b^WPR: within-person reliability.

^c^MOT: Multiple Object Tracking.

^d^Not available.

^e^DSM: Digit Symbol Matching.

^f^RTc: reaction time for correct responses.

^g^GradCPT: Gradual Onset Continuous Performance Test.

^h^d-prime: ability to discriminate targets from distractors.

^i^CRT: Choice Reaction Time.

### Number of EMAs Needed for Reliability

**Figure 3:**
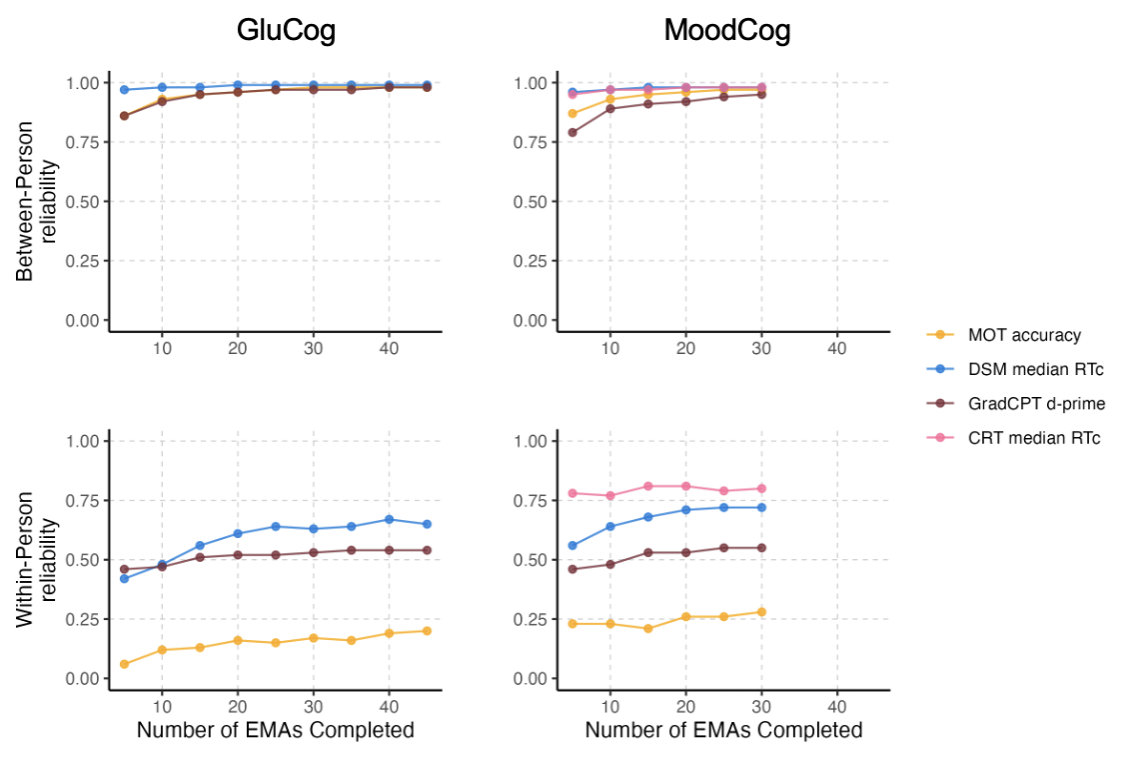
Within- and between-person reliabilities as functions of the number of ecological momentary assessments (EMAs) completed, separately for the GluCog and MoodCog groups. In the top-left panel, the line for Multiple Object Tracking (MOT) overlaps with the line for Gradual Onset Continuous Performance Test (GradCPT) such that only the GradCPT line is visible. CRT: Choice Reaction Time; d-prime: ability to discriminate targets from distractors; DSM; Digit Symbol Matching; RTc: reaction time responses.

Next, we analyzed between- and within-person reliability as a function of the completed number of cognitive EMAs (ie, how many assessments were needed to achieve maximum reliability) for the GluCog and MoodCog groups (Figure 3). For both the GluCog and MoodCog sample groups, maximum between-person reliability was observed after participants completed approximately 10 EMAs, with minimal improvements to reliability thereafter. Initially, we started with the first 5 EMAs and then computed the within-person reliability; after that, we took the first 10 EMAs and then computed the within-person reliability. This was to ensure that there were no radical changes over time and that, generally, the participants were performing and interacting with the test consistently over time.

When examining within-person reliability, the pattern was somewhat different between the 2 groups. In the GluCog sample, within-person reliability approached its maximum after the completion of 15 to 20 EMAs. In the MoodCog sample, the number of EMAs needed to achieve maximum within-person reliability varied by test, with some tests showing improvements in reliability up to approximately 15 EMAs (GradCPT and DSM) and others not showing such improvements (MOT and CRT). For both groups, across all tests, between-person and within-person reliability estimates were stable after 20 to 25 EMAs.

### Construct Validity of Cognitive EMA: Exploratory Factor Analysis

**Figure 4:**
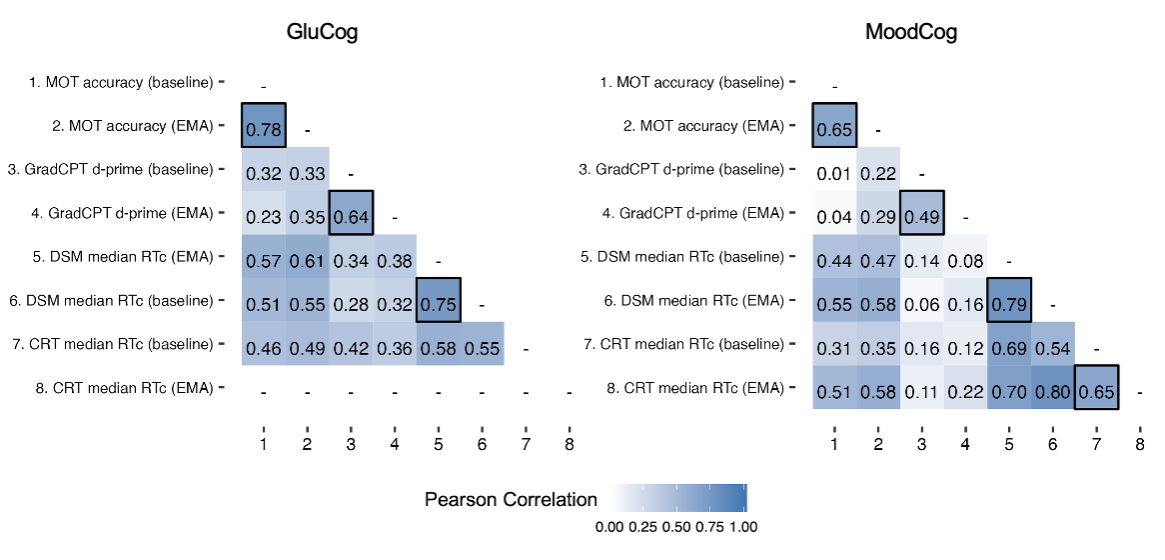
Correlation matrices (Pearson correlation) of the ecological momentary assessment (EMA) and full-length baseline versions of each cognitive test, separately for the GluCog and MoodCog groups. Correlations between the cognitive EMA and baseline versions of the tests are marked with a black border. CRT: Choice Reaction Time; d-prime: ability to discriminate targets from distractors; DSM: Digit Symbol Matching; GradCPT: Gradual Onset Continuous Performance Test; MOT: Multiple Object Tracking; RTc: reaction time for correct responses.

We assessed the construct validity of the EMA versions of the cognitive tests by examining the associations between the ultrabrief EMA cognitive tests and their corresponding full-length baseline versions. For both the GluCog and MoodCog samples, the ultrabrief EMA version of each cognitive test was generally more highly correlated with the corresponding full-length version of the same test than with the other tests (Figure 4).

Exploratory factor analysis was used to identify latent groupings among the ultrabrief cognitive EMA tests and their corresponding validated full-length baseline versions. Because the baseline EMAs were administered only once, we could not determine whether there were different factor loadings at the within-person level. For both samples (and as mentioned earlier), we conducted a parallel analysis [31] using the *fa.parallel*() function of the *psych* package in R [29]. This suggested 3 latent factors; therefore, we conducted an exploratory factor analysis with 3 latent factors and direct oblimin rotation (to allow for correlations between factors). On the basis of the resulting factor structure for each group, we identified 3 distinct factors corresponding to working memory (MOT only), attention (GradCPT only), and processing speed (DSM and CRT). The cumulative proportion of variance explained by these 3 factors was 0.72. The proportion of variance explained by each factor was 0.22 for working memory, 0.21 for sustained attention, and 0.29 for processing speed.

**Figure 5:**
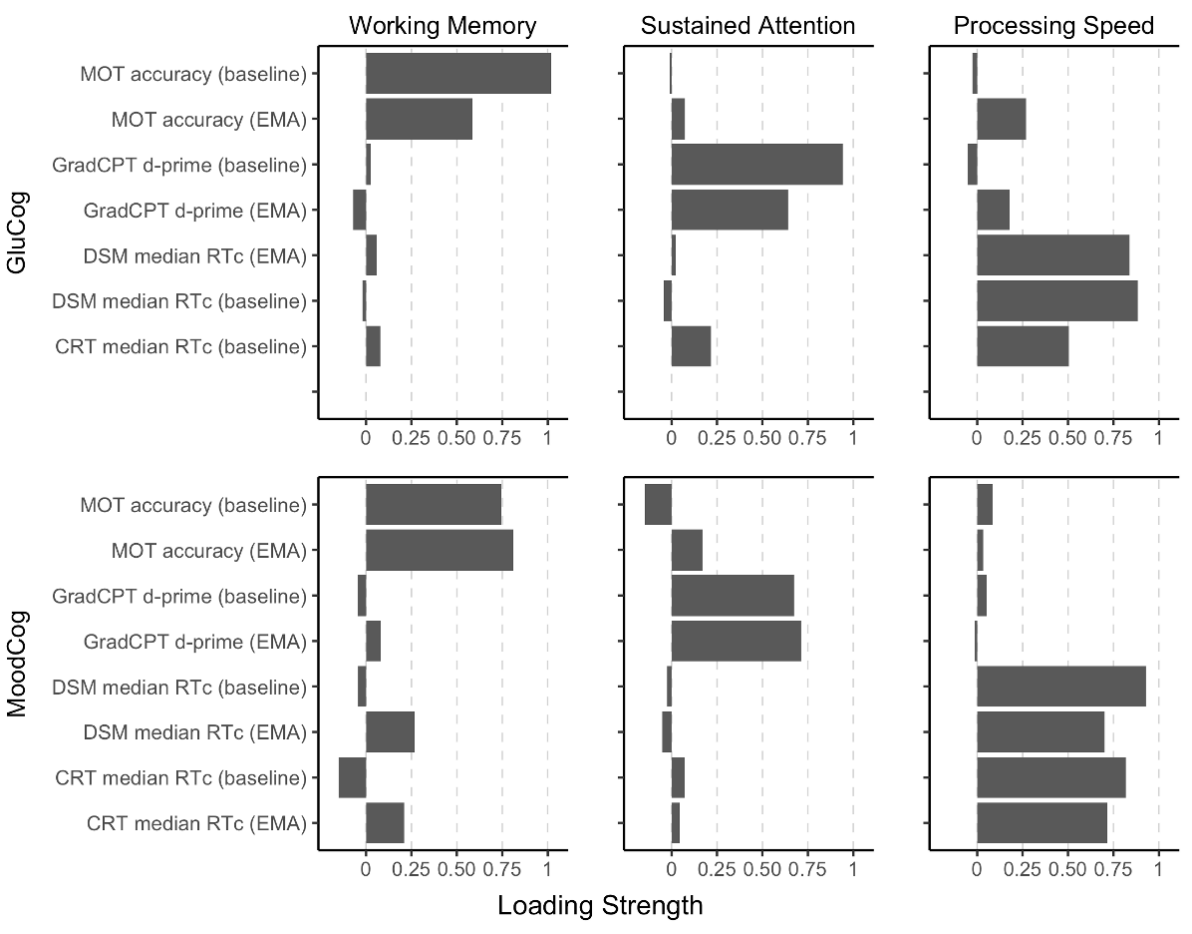
Factor structure of the ecological momentary assessment (EMA) and baseline versions of the cognitive EMA tests, separately for the GluCog (top) and MoodCog (bottom) groups. CRT: Choice Reaction Time; d-prime: ability to discriminate targets from distractors; DSM: Digit Symbol Matching; GradCPT: Gradual Onset Continuous Performance Test; MOT: Multiple Object Tracking; RTc: reaction time for correct responses.

Factor loadings and structure for each group are presented in Figure 5.

### Association With Age

**Figure 6:**
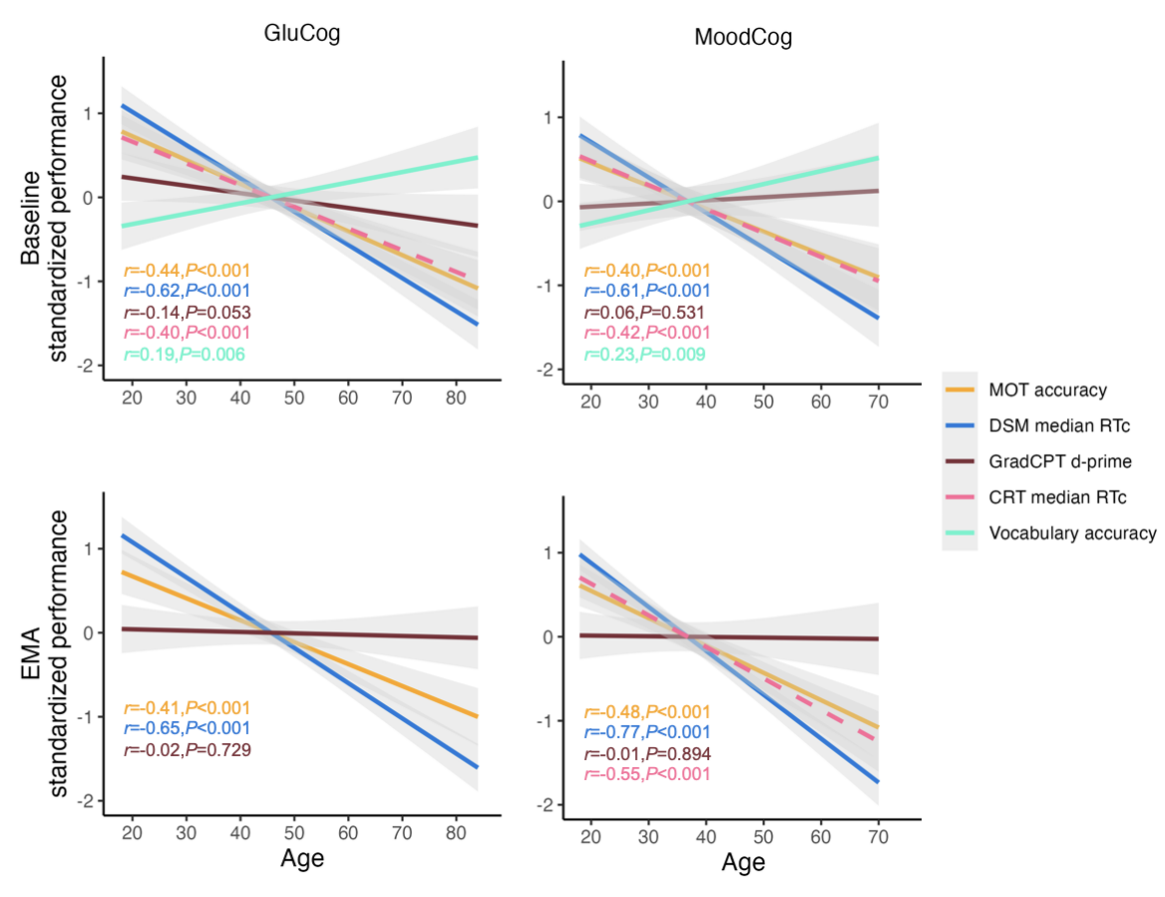
Relationships between cognitive test performance and age, separately for the GluCog and MoodCog groups. CRT: Choice Reaction Time; d-prime: ability to discriminate targets from distractors; DSM: Digit Symbol Matching; EMA: ecological momentary assessment; GradCPT: Gradual Onset Continuous Performance Test; MOT: Multiple Object Tracking; RTc: reaction time for correct responses.

Next, as an indicator of the validity for detecting differences across age, we analyzed whether cognitive test performance was correlated with age (Figure 6), as we would expect based on previous work [24,25]. As anticipated, older age was associated with worse working memory (poorer performance on full-length and EMA TMB MOT tasks; [32]) and slower processing speed (worse performance on full-length and ultrabrief EMA TMB DSM and TMB CRT tests; [24]). To confirm that older age was not associated with reduced performance because of reduced motivation or other more general issues not specific to these domains, we also examined the association between age and performance on the TMB Vocabulary Test. In addition, consistent with prior findings, older age was associated with better vocabulary performance [24]. Finally, we did not find a consistent linear association between age and sustained attention (full-length and EMA GradCPT d-prime). This is consistent with the prior findings that performance on this measure is stable across much of adulthood, with minimal decrements in performance until older age [25].

### Self-reported Interruptions

Finally, at the end of each EMA, we asked the participants to indicate whether they were interrupted during the completion of EMAs (yes or no). In both MoodCog and GluCog samples, the participants reported interruptions of some kind in approximately 25% of the EMAs. Specifically, the participants in the GluCog sample reported that an average of 10.8 (SD 8.10) of their 45 (24.1%) EMAs were interrupted, and the participants in the MoodCog sample reported that an average of 7.50 (SD 5.40) of their 30 (25%) EMAs were interrupted.

**Figure 7:**
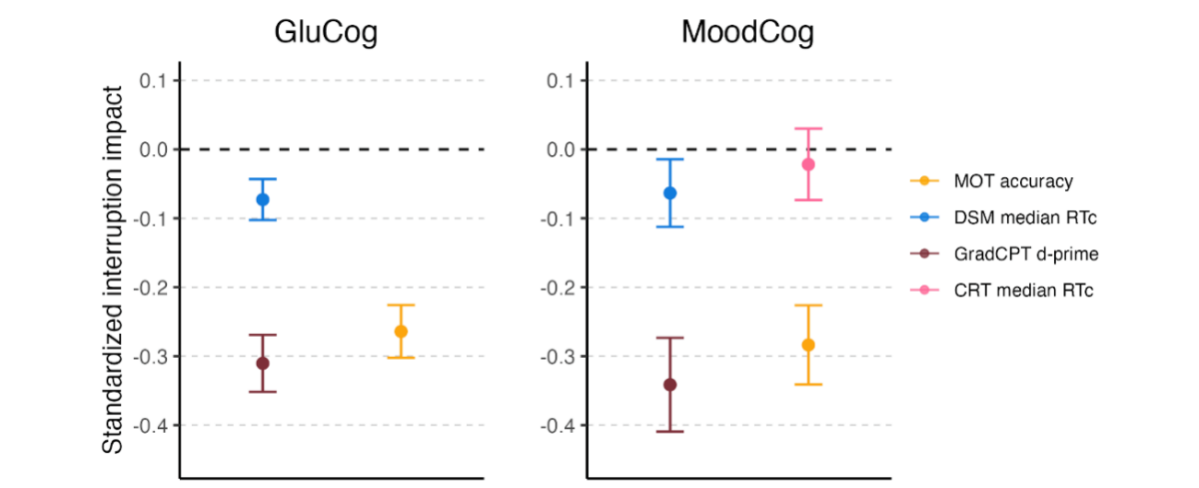
Impact of interruptions on momentary cognitive performance, separately for the GluCog and MoodCog groups. The points illustrate the estimates of the impact of interruptions in standardized units, and the error bars indicate the corresponding CIs. Negative values indicate a high impact on test performance. CRT: Choice Reaction Time; d-prime: ability to discriminate targets from distractors; DSM: Digit Symbol Matching; GradCPT: Gradual Onset Continuous Performance Test; MOT: Multiple Object Tracking; RTc: reaction time for correct responses.

For both samples, self-reported interruptions were associated with worse working memory (TMB MOT: GluCog β=−.26; *P*<.001; MoodCog β=−.28; *P*<.001) and worse sustained attention (TMB GradCPT: GluCog β=−.31; *P*<.001; MoodCog β=−.34; *P*<.001). Interruptions were also associated with a slower median reaction time for TMB DSM in both samples, but to a smaller degree (TMB DSM: GluCog β=−.07; *P*<.001; MoodCog β=−.06; *P*<.01). Interruptions were not related to differences in performance in TMB CRT (MoodCog: β=−.02; *P*=.41). This is likely because the median reaction time metric is relatively unaffected by outlier values, whereas the outcome metrics for working memory and sustained attention aggregate across all trials (Figure 7).

## Discussion

### Principal Findings

#### Overview

Cognitive EMA represents a novel approach to evaluating fluctuations in cognitive status over time. The completion rates in both studies demonstrate that cognitive EMA can be feasibly used in both a clinical sample of individuals with high levels of expected cognitive variability recruited through clinics and a community sample of individuals with normative levels of cognitive variability recruited on the web (through our digital research platform, TMB [17]). To our knowledge, this study is the first to report the psychometrics of cognitive EMA in clinical and community samples. Other prior studies [33] examining community samples used multiple time points over longer periods, whereas our study had a higher-frequency burst design, requiring multiple time points of assessments. Currently, neuropsychologists and others measuring cognition are limited in the scope of assessment, typically capturing 1 to 2 time points or a “snapshot” of a patient’s functioning. In terms of the goals of our study, which were to demonstrate the reliability and validity of ultrabrief cognitive EMAs in clinical and community samples, our findings suggest that there is an optimal number of EMAs with good reliability, consistent validity markers, and specific tests that are more and less robust to contextual factors, such as interruptions during test administration, that can be administered in these samples.

#### Reliability

To better capture the psychometric properties of these tests, we evaluated both between- and within-person reliabilities. EMA uniquely allows the tracking of fluctuations in a single participant’s functioning over repeated assessments (within-person measurement) while simultaneously improving our ability to measure an individual’s performance relative to others (traditional, between-person measurement). Estimating between-person reliability allows us to examine the stability of this between-person assessment, that is, it demonstrates the reliability of the differences between participants aggregated across testing sessions. Within-person reliability, by contrast, provides the consistency of scores between time points (within participants) or the reliability of the performance differences evaluated between ≥2 testing sessions. In most of neuropsychology, the type of reliability reported when evaluating the psychometric characteristics of a test is between-person reliability. In our study, we found the expected high between-person reliability across all tests. Within-person reliability values were more modest for the cognitive EMA measures we evaluated, with poor within-person reliability for visuospatial working memory (TMB MOT) and good within-person reliability for measures related to processing speed. Low within-person reliability for MOT may have been due to the variation in scores over time within individuals. This is consistent with the findings from the study by Sliwinski et al [6], which demonstrated that within-person reliability tends to be lower in accuracy-based tests and that there can be significant scores from test to test. In the case of MOT, which is an accuracy-based test, performance tends to be highly influenced by participants’ guessing choices. A key difference between within-person reliability and traditional test-retest reliability, with all else being equal, is that reducing within-person variability increases test-retest reliability (as scores are more stable over time) but reduces within-person reliability, which depends on cognitive variability over time within participants. Thus, for tests where scores are stable or robust to differences in stress, physiology, fatigue, or context, within-person reliability is limited by a lack of within-person variability. In the existing literature, the within-person reliability of cognitive tests is typically lower than the between-person reliability of high-frequency cognitive testing protocols (eg, measurement burst designs), likely because many cognitive tests were designed to capture between-person differences. Our reliability values for both between- and within-person reliabilities were comparable with those obtained for high-frequency cognitive testing in a measurement burst design in an older adult sample [6].

The GluCog and MoodCog groups completed a similar proportion of total EMAs (approximately 85%). In line with previous findings [6], tests in both groups established strong between-person reliability after completing 10 EMAs (previous study established that those EMAs were completed over 3 days). Within-person reliability, although generally stable regardless of the number of EMAs completed, varied based on the test; processing speed tests had the highest within-person reliability, followed by the sustained attention and working memory tasks. Tests with higher within-person reliability are likely to be well suited for identifying factors that may impact cognitive functioning over short time intervals. Clinically, these factors are important when evaluating cognitive impairment, particularly when determining whether there is a bona fide decline in cognitive functioning or intratest variability. For interventions aimed at maximizing cognitive performance by reducing the modifiable causes of cognitive impairment (eg, poor sleep and low mood), the presence of variation within person over time may uncover important treatment targets.

#### Validity

The full-length baseline and ultrabrief EMA versions of the cognitive tests correlated highly with one another and loaded together as expected when using an exploratory factor analysis approach. As expected based on prior literature evaluating these cognitive tests (eg, Singh et al [34]), for both the GluCog and MoodCog groups, both versions of the MOT task mapped onto working memory, both versions of the GradCPT mapped onto attention, and both versions of the DSM test and the baseline version of CRT mapped onto processing speed. For the MoodCog group that completed the ultrabrief EMA version of CRT, the ultrabrief EMA version of CRT also mapped onto processing speed. All tests also varied with age, as expected based on previous research [24]. In both samples, the scores on the tests of processing speed and visuospatial working memory declined sharply with age, based on both full-length and ultrabrief EMA measures. Sustained attention scores were relatively stable across the age range for both samples [25]. At the same time, TMB vocabulary scores (based on baseline, full-length assessment) improved across the age range for both samples. Thus, the EMA versions of these tests were judged to be valid with respect to the expected age-related differences.

#### Interruptions

The participants in both MoodCog and GluCog groups were provided with a 30-minute window to complete EMAs, with the direction to complete the assessments at a time when disruptions would be minimal. Nevertheless, the participants reported interruptions (in response to a post-EMA self-report question) at approximately 25% of the time points. As expected and supporting the use of interruptions as a validity marker, interruptions were related to poorer performance on the ultrabrief cognitive EMA. This is important to account for when designing EMA studies, particularly given that EMAs are completed in natural environments. On the one hand, if aiming to achieve diagnostic accuracy (ie, inferring underlying brain pathology), it may benefit future investigations using cognitive EMA to optimize the delivery timing of EMAs to ensure that delivery occurs when participants are less likely to be interrupted (early morning or evening). However, if the goal of EMA assessment is to understand how patients function in their everyday lives, measuring the impact of interruptions on cognitive performance may have important clinical implications. The results showed that different tests and cognitive domains may be more robust versus sensitive to interruptions. Specifically, the scores on accuracy-based tests were more impacted by interruptions than the scores on reaction time–based tests (where scores were estimated using the median). For evaluating individuals who may have cognitive impairments that are more transient or sensitive to interruptions, it is likely that the TMB MOT test and GradCPT would be best at capturing momentary cognitive deficits. For cases where there is a need for measures that are more resistant to interruptions, based on our findings, we recommend processing speed tests such as DSM and CRT and using test scores that are more robust to the influence of outliers (eg, the use of median rather than mean reaction times).

### Limitations

Although we demonstrated that cognition in these 2 samples can be measured reliably, this study was not designed to characterize the source and context of this variability. For example, although we measured interruptions and related them to poorer cognitive functioning, we did not have contextual information to further characterize the nature and severity of the interruptions (eg, hearing a loud noise but being able to continue vs stopping the test to talk to someone). Given the concern about participant burden and the necessarily brief nature of EMA, this study is limited in the number of cognitive tests and domains that were evaluated, which limits the generalizability of our findings. In addition, these cognitive EMAs were administered in an uncontrolled environment, which is different from the way cognitive tests are administered in clinics (ie, in highly controlled environments); however, neuropsychology and neuropsychological research are increasingly moving toward digital and more naturalistic approaches to cognitive testing that must account for these sources of variability [35]. Thus, as with any study, researchers need to weigh the tradeoffs between single time point, longer, and controlled assessments and more frequent, shorter EMAs in naturalistic settings, noting that, as discussed earlier, naturalistic assessments can indeed complement in-clinic assessments without necessarily replacing them. Regarding the tests themselves, the ultrabrief cognitive EMAs used in this study were validated based on baseline (full-length) TMB cognitive tests instead of the traditional paper-and-pencil tests typically administered in neuropsychological evaluations. In this study, we did not have sufficient power to analyze within- and between-person reliability stratified by other demographic variables, such as education; we chose age as a meaningful variable to associate with cognitive test performance based on its association with cognitive performance in previous research [24]. In terms of gathering self-report data, we asked the participants to self-report diseases and drug use; this presents its own set of limitations in that we cannot corroborate self-reports with previous medical records.

### Future Directions

In future studies, we aim to analyze the predictors of within-person variation to identify key factors that substantially impact fluctuations in cognitive status. This would include the analyses of physiological variables such as glucose and psychological factors such as mood and symptom severity. By understanding the factors that most substantially contribute to variability in cognitive status, we can begin to generate insights that help address fluctuations in cognitive status in clinical populations. Future studies may also further validate the use of ultrabrief cognitive EMAs in clinical samples by associating the cognitive performance on these tasks with the results of traditional paper-and-pencil tests typically administered in neuropsychological evaluations. This would facilitate implementation in traditional clinical workflows, which would provide additional opportunities for research focused on patient outcomes.

### Conclusions

This study examined the between- and within-person reliabilities and validity of the EMA versions of cognitive tests in clinical and community samples. Analyses demonstrated that there was strong between-person reliability after the completion of approximately 10 EMAs in both samples. Although within-person reliability was relatively stable, it was generally higher for tests of processing speed than for tests of sustained attention and working memory in both clinical and community participants. Furthermore, approximately 25% of the completed EMAs were subject to (self-reported) interruptions, which decreased performance on measures of working memory and sustained attention but was less important for processing speed. In general, cognitive EMA offers substantial benefits, as it permits the collection of rich between- and within-person data that can improve the monitoring of cognitive functioning. Future studies will aim to understand which internal and external factors best account for within-person variability in cognitive status.

## Appendix

Multimedia Appendix 1 Additional data from the GluCog and MoodCog samples.

## Abbreviations

CRT: Choice Reaction Time
DSM: Digit Symbol Matching
EMA: ecological momentary assessment
GradCPT: Gradual Onset Continuous Performance Test
IRB: institutional review board
MOT: Multiple Object Tracking
T1D: type 1 diabetes
TMB: TestMyBrain
WAIS-III: Wechsler Adult Intelligence Scale-Third Edition
WAIS-IV: Wechsler Adult Intelligence Scale-Fourth Edition

## References

[1] Germine L, Strong RW, Singh S, Sliwinski MJ (2021). Toward dynamic phenotypes and the scalable measurement of human behavior. Neuropsychopharmacology.

[2] Wilks H, Aschenbrenner AJ, Gordon BA, Balota DA, Fagan AM, Musiek E, Balls-Berry J, Benzinger TL, Cruchaga C, Morris JC, Hassenstab J (2021). Sharper in the morning: cognitive time of day effects revealed with high-frequency smartphone testing. J Clin Exp Neuropsychol.

[3] Chaytor N, Schmitter-Edgecombe M, Burr R (2006). Improving the ecological validity of executive functioning assessment. Arch Clin Neuropsychol.

[4] Sliwinski MJ, Smyth JM, Hofer SM, Stawski RS (2006). Intraindividual coupling of daily stress and cognition. Psychol Aging.

[5] Allard M, Husky M, Catheline G, Pelletier A, Dilharreguy B, Amieva H, Pérès K, Foubert-Samier A, Dartigues JF, Swendsen J (2014). Mobile technologies in the early detection of cognitive decline. PLoS One.

[6] Sliwinski MJ, Mogle JA, Hyun J, Munoz E, Smyth JM, Lipton RB (2018). Reliability and validity of ambulatory cognitive assessments. Assessment.

[7] Spooner DM, Pachana NA (2006). Ecological validity in neuropsychological assessment: a case for greater consideration in research with neurologically intact populations. Arch Clin Neuropsychol.

[8] Kaya Y, Bostan S, Kaya A, Karaman Ö, Karataş A, Dereli S (2021). Effect of COVID-19 pandemic on anxiety depression and intention to go to hospital in chronic patients. Int J Clin Pract.

[9] Singh S, Germine L (2021). Technology meets tradition: a hybrid model for implementing digital tools in neuropsychology. Int Rev Psychiatry.

[10] Nicosia J, Aschenbrenner AJ, Adams SL, Tahan M, Stout SH, Wilks H, Balls-Berry JE, Morris JC, Hassenstab J (2022). Bridging the technological divide: stigmas and challenges with technology in digital brain health studies of older adults. Front Digit Health.

[11] Cerino ES, Katz MJ, Wang C, Qin J, Gao Q, Hyun J, Hakun JG, Roque NA, Derby CA, Lipton RB, Sliwinski MJ (2021). Variability in cognitive performance on mobile devices is sensitive to mild cognitive impairment: results from the Einstein aging study. Front Digit Health.

[12] Smyth JM, Stone AA (2003). Ecological momentary assessment research in behavioral medicine. J Happiness Stud.

[13] Shiffman S, Stone AA, Hufford MR (2008). Ecological momentary assessment. Annu Rev Clin Psychol.

[14] Hyun J, Sliwinski MJ, Smyth JM (2019). Waking up on the wrong side of the bed: the effects of stress anticipation on working memory in daily life. J Gerontol B Psychol Sci Soc Sci.

[15] Brands AM, Biessels GJ, de Haan EH, Kappelle LJ, Kessels RP (2005). The effects of type 1 diabetes on cognitive performance: a meta-analysis. Diabetes Care.

[16] Mõttus R, Luciano M, Starr JM, Deary IJ (2013). Diabetes and life-long cognitive ability. J Psychosom Res.

[17] TestMyBrain.

[18] Mascarenhas Fonseca L, Strong RW, Singh S, Bulger JD, Cleveland M, Grinspoon E, Janess K, Jung L, Miller K, Passell E, Ressler K, Sliwinski MJ, Verdejo A, Weinstock RS, Germine L, Chaytor NS (2023). Glycemic variability and fluctuations in cognitive status in adults with type 1 diabetes (GluCog): observational study using ecological momentary assessment of cognition. JMIR Diabetes.

[19] Hawks ZW, Strong R, Jung L, Beck ED, Passell EJ, Grinspoon E, Singh S, Frumkin MR, Sliwinski M, Germine LT (2022). Accurate prediction of momentary cognition from intensive longitudinal data. Biol Psychiatry Cogn Neurosci Neuroimaging (forthcoming).

[20] Treviño M, Zhu X, Lu YY, Scheuer LS, Passell E, Huang GC, Germine LT, Horowitz TS (2021). How do we measure attention? Using factor analysis to establish construct validity of neuropsychological tests. Cogn Res Princ Implic.

[21] Pylyshyn ZW, Storm RW (1988). Tracking multiple independent targets: evidence for a parallel tracking mechanism. Spat Vis.

[22] Wechsler D (1997). Wechsler Adult Intelligence Scale. 3rd edition.

[23] Chaytor NS, Barbosa-Leiker C, Germine LT, Fonseca LM, McPherson SM, Tuttle KR (2021). Construct validity, ecological validity and acceptance of self-administered online neuropsychological assessment in adults. Clin Neuropsychol.

[24] Hartshorne JK, Germine LT (2015). When does cognitive functioning peak? The asynchronous rise and fall of different cognitive abilities across the life span. Psychol Sci.

[25] Fortenbaugh FC, DeGutis J, Germine L, Wilmer JB, Grosso M, Russo K, Esterman M (2015). Sustained attention across the life span in a sample of 10,000: dissociating ability and strategy. Psychol Sci.

[26] Esterman M, Noonan SK, Rosenberg M, Degutis J (2013). In the zone or zoning out? Tracking behavioral and neural fluctuations during sustained attention. Cereb Cortex.

[27] Rutter LA, Vahia IV, Forester BP, Ressler KJ, Germine L (2020). Heterogeneous indicators of cognitive performance and performance variability across the lifespan. Front Aging Neurosci.

[28] Germine LT, Joormann J, Passell E, Rutter LA, Scheuer L, Martini P, Hwang I, Lee S, Sampson N, Barch DM, House SL, Beaudoin FL, An X, Stevens JS, Zeng D, Linnstaedt SD, Jovanovic T, Clifford GD, Neylan TC, Rauch SL, Lewandowski C, Hendry PL, Sheikh S, Storrow AB, Musey PI, Jones CW, Punches BE, McGrath ME, Pascual JL, Mohiuddin K, Pearson C, Peak DA, Domeier RM, Bruce SE, Rathlev NK, Sanchez LD, Pietrzak RH, Pizzagalli DA, Harte SE, Elliott JM, Koenen KC, Ressler KJ, McLean SA, Kessler RC (2022). Neurocognition after motor vehicle collision and adverse post-traumatic neuropsychiatric sequelae within 8 weeks: initial findings from the AURORA study. J Affect Disord.

[29] Revelle W (2022). psych: procedures for psychological, psychometric, and personality research. R package version 2.2.5. The Comprehensive R Archive Network.

[30] Revelle W, Wilt J (2019). Analyzing dynamic data: a tutorial. Pers Individ Dif.

[31] Zwick WR, Velicer WF (1986). Comparison of five rules for determining the number of components to retain. Psychol Bull.

[32] Trick LM, Jaspers-Fayer F, Sethi N (2005). Multiple-object tracking in children: the “catch the spies” task. Cogn Dev.

[33] Brown LJ, Adlam T, Hwang F, Khadra H, Maclean LM, Rudd B, Smith T, Timon C, Williams EA, Astell AJ (2016). Computer-based tools for assessing micro-longitudinal patterns of cognitive function in older adults. Age (Dordr).

[34] Singh S, Strong RW, Jung L, Li FH, Grinspoon L, Scheuer LS, Passell EJ, Martini P, Chaytor N, Soble JR, Germine L (2021). The TestMyBrain digital neuropsychology toolkit: development and psychometric characteristics. J Clin Exp Neuropsychol.

[35] Parsons T, Duffield T (2020). Paradigm shift toward digital neuropsychology and high-dimensional neuropsychological assessments: review. J Med Internet Res.

