# Supplementary material for: Ecological Momentary Assessment of Cognition in Clinical and Community Samples: Reliability and Validity Study
Source: J Med Internet Res. 2023 Jun 2;25:e45028. doi: 10.2196/45028 (PMC10276323; doi:10.2196/45028)
Supplement: Multimedia Appendix 1 [file jmir_v25i1e45028_app1.docx]

**SUPPLEMENT**

Table S1. Withdrawal Procedure for the GluCog and MoodCog Groups.

|  | GluCog | MoodCog |
| --- | --- | --- |
| Enrolled in the study &  Did not withdraw due to technical issues or voluntarily | 203 | 156 |
| Withdrawn involuntarily  GluCog: completion of less than half of EMAs by day 7;  MoodCog: missing 6 consecutive EMAs | 2 | 28 |
| Completed study | 201 | 128 |
| Completed study & Completed at least 50% EMAs | 198 | 128 |

Table S2. Quality Control Criteria for EMA Exclusion

| Test Name | EMA Exclusion Criteria | EMA Excluded N (%) | |
| --- | --- | --- | --- |
|  |  | GluCog | MoodCog |
| Multiple Object Tracking | Frametime > 30 milliseconds | 367 (4.8) | 174 (4.5) |
| Digit Symbol Matching | Accuracy < 50% OR  Correct responses < 6 | 134 (1.8) | 21 (0.7) |
| Gradual Onset Continuous Performance Test | Omission rate >= 50% | 157 (2.1) | 43 (1.3) |
| Choice Reaction Time | Accuracy <= 60% | - | 34 (1.1) |

Note. Frametime that exceeded 30 milliseconds indicates the device was in a power save mode and the animation of moving dots was not displayed as intended.
